# Low Transmission of Chikungunya Virus by *Aedes aegypti* from Vientiane Capital, Lao PDR

**DOI:** 10.3390/pathogens12010031

**Published:** 2022-12-25

**Authors:** Elodie Calvez, Elliott F. Miot, Sitsana Keosenhom, Vaekey Vungkyly, Souksakhone Viengphouthong, Phaithong Bounmany, Paul T. Brey, Sébastien Marcombe, Marc Grandadam

**Affiliations:** 1 Arbovirus and Emerging Viral Diseases Laboratory, Institut Pasteur du Laos, Vientiane 01030, Laos; 2 Laboratory of Vector Control Research, Unit Transmission Reservoir and Pathogens Diversity, Institut Pasteur de la Guadeloupe, 97139 Les Abymes, France; 3 Medical Entomology and Vector-Borne Disease Unit, Institut Pasteur du Laos, Vientiane 01030, Laos; 4 State Key Laboratory of Emerging Infectious Diseases, School of Public Health, The University of Hong Kong, Hong Kong, China; 5 Centre for Immunology & Infection Limited, Hong Kong, China; 6 HKU—Pasteur Research Pole, The University of Hong Kong, Hong Kong, China; 7 Institut de Recherche Biomédicale des Armées, 91220 Brétigny-sur-Orge, France

**Keywords:** Chikungunya virus, *Aedes aegypti*, vector competence, Lao PDR

## Abstract

In 2012–2013, chikungunya virus (CHIKV) was the cause of a major outbreak in the southern part of Lao People’s Democratic Republic (Lao PDR). Since then, only a few imported cases, with isolates belonging to different lineages, were recorded between 2014 and 2020 in Vientiane capital and few autochthonous cases of ECSA-IOL lineage were detected in the south of the country in 2020. The CHIKV epidemiological profile contrasts with the continuous and intensive circulation of dengue virus in the country, especially in Vientiane capital. The study’s aim was to investigate the ability of the local field-derived *Aedes aegypti* population from Vientiane capital to transmit the Asian and ECSA-IOL lineages of CHIKV. Our results revealed that, for both CHIKV lineages, infection rates were low and dissemination rates were high. The transmission rates and efficiencies evidenced a low vector competence for the CHIKV tested. Although this population of *Ae. aegypti* showed a relatively modest vector competence for these two CHIKV lineages, several other factors could influence arbovirus emergence such as the longevity and density of female mosquitoes. Due to the active circulation of CHIKV in Southeast Asia, investigations on these factors should be done to prevent the risk of CHIKV emergence and spread in Lao PDR and neighboring countries.

## 1. Introduction

Chikungunya virus (CHIKV; *Togaviridae*, genus *Alphavirus*) is a mosquito-borne virus, transmitted to humans through the bite of the *Aedes* mosquito, especially *Ae. aegypti* and *Ae. albopictus* within urban and peri-urban cycles [1,2,3]. Various intrinsic and extrinsic factors can influence the dynamics of arbovirus transmission by mosquitoes, such as vector competence, survival, density, biting rate, and duration of the extrinsic incubation period [4,5]. Chikungunya fever syndrome in humans includes high fever, headache, and maculopapular rash, but more specifically, severe and incapacitating joint pains that may evolve into chronic polyarthralgia [6]. CHIKV is an enveloped single-stranded RNA positive-sense virus of 11.7 kb [7]. Previous phylogenetic studies have determined four distinct lineages of CHIKV strains: the West African (WA), East/Central/South African (ECSA), ECSA-derived Indian Ocean (ECSA-IOL), and Asian lineage [8,9].

Since the first CHIKV isolation in Uganda in 1952 [10,11], the virus has been detected in many sub-Saharan African countries, in Asia, and more recently in the Americas, South Pacific, and Europe [8,12,13,14,15]. In Asia, CHIKV was first reported in 1958 in Thailand, but retrospective studies of human sera suggest that the virus has circulated in the region prior the discovery of the virus [15,16]. Since then, in addition to the former Asian lineage, two additional lineages have emerged and spread in this part of the world since 2006: ECSA and ECSA-IOL [15,16,17,18,19].

Lao People’s Democratic Republic (Lao PDR) is a low-income country located in Southeast Asia and occupies a central position within the Indochinese peninsula. This geographical location and the presence of primary arbovirus vectors such as *Ae. aegypti* and *Ae. albopictus* increase the risk of arbovirus circulation, such as CHIKV and dengue viruses (DENV), in the country [20,21,22,23,24,25]. Unfortunately, little is known about the actual ability of these vectors to transmit arboviruses. Since 2012, an integrated arbovirus surveillance network was set up by the Institut Pasteur du Laos [20]. This surveillance system, combining laboratory capacities and field entomologic studies (vector repartition and insecticide resistance studies), provides major data on the circulation of DENV serotypes, CHIKV, and Zika viruses [20,22,26], and evidenced multiple-insecticide resistance profiles of local *Ae. aegypti* and *Ae. albopictus* populations [24,25]. In 2012–2013, a CHIKV outbreak, caused by the ECSA-IOL lineage, spread out in Champasak Province and remained limited to southern Lao provinces [23,27,28].

Previous data from the 1970s evidenced the presence of anti-CHIKV antibodies in 30% of the general population in Vientiane capital [29]. A more recent study was held in the capital but all the samples tested (n = 200) were found negative for CHIKV ELISA and RT-PCR [27]. These results contrasted with the active circulation and co-circulation of the four DENV serotypes in this city since 1979 [20,21,22]. The risk of CHIKV and DENV transmission is potentiated by the presence of both *Ae. aegypti* and *Ae. albopictus*. Entomological data, collected since 2016, showed that these vectors represented, respectively, 86% and 14% of *Aedes* mosquitoes collected in Vientiane city [20]. Thus, the risk of the re-introduction and spread of CHIKV in Vientiane capital and the rest of the country persists. However, the chances of a successful re-emergence of the virus may vary depending on, at least in part, viral genetic features, mosquito species, and the vector populations’ ability to be infected and to transmit the virus.

The aim of this study was to evaluate the experimental ability of the *Ae. aegypti* population from Vientiane, the predominant possible vector for CHIKV, to transmit different lineages of the virus to improve our knowledge on virus–vector interaction in Lao PDR. For this purpose, we performed experimental infections with a local and an imported CHIKV isolate belonging to ECSA-IOL and Asian lineages, respectively, on an *Ae. aegypti* population from Vientiane capital city.

## 2. Materials and Methods

### 2.1. Ethics Statement

Human sample collection and laboratory procedures for the arbovirus surveillance program held by the Institut Pasteur du Laos have been approved by the Lao Ministry of Health‘s National Ethic Committee for Health Research (N°114/NECHR). Plasma samples used in this study were previously obtained from anonymized patients who were unopposed to the secondary use of their biological material for research on arboviruses. This study follows Lao PDR Animal Ethics Guidelines.

### 2.2. Virus Strains

The two CHIKV strains used in this study were obtained from patients diagnosed in Lao PDR. The first strain (H2013-445) was isolated during the 2013 CHIKV outbreak in Pakse, Champasak province and belonged to the ECSA-IOL lineage, Asian sub-lineage (GenBank: LN901348) [23]. The second strain (H2019-9293) was isolated in 2019 in Vientiane capital, from a patient coming back from Indonesia. This strain belonged to the Asian lineage (E2-6K-E1 region; GenBank: MZ292729) [30]. Both strains presented the E1-A226V mutation, a signature of the increased transmission of CHIKV by *Ae. albopictus* [31]. The final viral stocks were prepared after two passages in mammalian Vero E6 cells maintained in Medium 199 (Gibco™, Thermo Fisher Scientific, Waltham, MA, USA) supplemented with 2% fetal bovine serum (FBS; Gibco™, Thermo Fisher Scientific, Waltham, MA, USA). After three days of incubation at 37 °C with 5% CO_2_, the supernatants were collected and stored at −80 °C. Viral titers were determined using serial 10-fold dilution on Vero E6 cells and expressed as TCID_50_/mL.

### 2.3. Mosquito Collections

The original *Ae. aegypti* specimens were collected at the immature stage from four traps located in Sivilay village, Vientiane capital, Lao PDR (18.010516° N, 102.632912° E) in March 2019. The larvae and pupae were reared under controlled laboratory conditions (27 ± 2 °C, 80% relative humidity, and 12:12 h light–dark cycle) with permanent access to 10% sucrose solution. The females were fed several times with fresh pig blood obtained from a local slaughterhouse, supplemented with 10 mM adenosine triphosphate (Merck, Darmstadt, Germany), through a capsule (Hemotek system, Discovery Workshops, Accrington, UK) covered by a feeding membrane made of a fragment of pig intestine membrane, freshly obtained also from a slaughterhouse. For infection assays, F3 were hatched, and the adults were maintained as described above.

### 2.4. Mosquito Oral Infections

Experimental mosquito infections were performed in a BSL-3 facility. For each virus, 3–4 boxes of 60 mosquito females aged from four to seven days old, not previously blood-fed, were starved for 24 h before infection. The female mosquitoes were allowed to take an infectious blood meal made of a mix of 2 mL of washed pig erythrocytes and 1 mL of viral suspension adjusted to 10^6^ TCID_50_/mL and supplemented with 10 mM adenosine triphosphate as a phagostimulant [32], dispensed through the Hemotek system as described above. After 20 min, the blood meal was interrupted, and the fully engorged females were transferred into new containers and maintained at 30 ± 2 °C and 70 ± 5% relative humidity under a 12:12 h light–dark cycle (Memmert climate chamber, Memmert GmbH + Co.KG, Schwabach, Germany) with permanent access to 10% sucrose solution.

### 2.5. Infection, Dissemination, and Transmission Analysis

Groups of 28–30 female mosquitoes were randomly collected at days 3, 7, and 14 after the infectious blood meal, and cold-anesthetized. The legs and wings of each mosquito were carefully removed, and the proboscis was inserted into a filter tip containing 5 µL of FBS for 30 min. After the salivation, the 5 µL harvested were added to 45 µL of Medium 199 1×. The mosquito heads and bodies were separated and stored in individual tubes, carefully labelled. All samples were stored at −80 °C.

For each mosquito, the body, head, and saliva were sampled to determine, respectively, the infection, dissemination, and transmission rates. The bodies and heads were individually ground in 250 µL of Medium 199 1× supplemented with 2% FBS and antibiotics/antifungals (100 units/mL of penicillin, 0.1 mg/mL of streptomycin, and 0.25 µg/mL amphotericin B). Lysis was carried out in a TissueLyser (Qiagen, Hilden, Germany) set for 2 min at 30 Hz. The samples were centrifuged at 6000 rpm for 10 min at 4 °C. The body and head supernatants were stored at −80 °C before analysis. To determine infection and dissemination rates, viral RNA was extracted from each body and head samples using the NucleoSpin RNA virus kit (Macherey-Nagel, Düren, Germany) according to the manufacturer’s instructions. The presence of viral particles was determined by real-time reverse transcription polymerase chain reaction (RT-PCR) using the primers previously described [33]. For CHIKV particle detection, 20 µL of saliva suspension was inoculated onto Vero E6 cells in 24-wells plates and incubated at 37 °C for five days. The presence of infectious particles was assessed by the detection of the cytopathic effect (CPE).

### 2.6. Statistical Analysis

Infection rate (IR; the number of positive bodies divided by the total number of mosquitoes tested), dissemination rate (DR; the number of infected heads divided by the number of infected bodies), transmission rate (TR; the number of infected saliva samples divided by the number of infected heads), and transmission efficiency (TE; the number of virus-positive saliva samples divided by the total number of mosquitoes tested) were calculated for each CHIKV strain at each day post-infection (dpi).

Vector competence indices (i.e., IR, DR, TR, and TE) were analyzed with a logistic regression model with each individual mosquito associated with a binary variable (1 = CHIKV-positive, or 0 = CHIKV-negative), followed by an analysis of deviance with the *car* package [34]. The models included the effect of the viral strain (H2013-445 or H2019-9293), the day post-infection (3-, 7-, or 14-dpi), and their interaction. For TR, only 7- and 14-dpi for both strains were included in the model since no dissemination was observed at 3-dpi for H2013-445, and therefore there was no TR value. A comparison between the conditions was performed using a multiple comparison of means followed by Tukey’s post-hoc test using the *multcomp* package [35]. All statistical analyses were performed with R v. 4.0.5 (R Core Team, Vienna, Austria) [36], and graphical representations were generated using the R packages *ggplot2* and *plyr* [37,38].

## 3. Results

To evaluate the ability of *Ae. aegypti* from Lao PDR to transmit CHIKV, a mosquito population from Vientiane capital was independently infected with two strains of CHIKV belonging to the ESCA-IOL and Asian lineages at an infectious dose of 10^6^ TCID_50_/mL.

**Figure 1:**
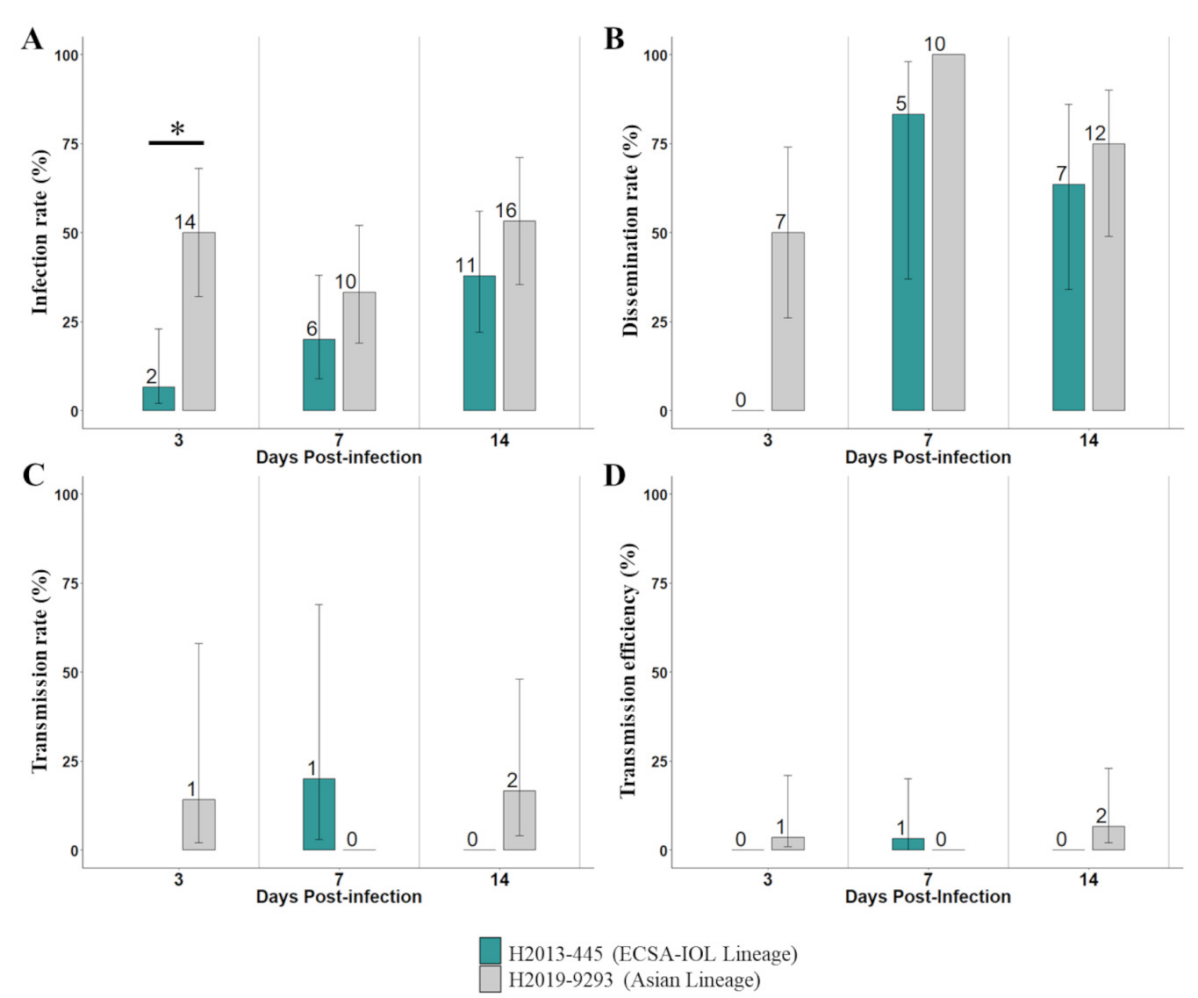
Experimental infections of Aedes aegypti from Lao PDR with CHIKV belonging to the ECSA-IOL and Asian lineages. (**A**) Infection rate, the proportion of blood-fed mosquitoes with an infected body; (**B**) dissemination rate, the proportion of infected mosquitoes with virus disseminated to the head tissues; (**C**) transmission rate, the proportion of mosquitoes with a disseminated infection presenting virus in their saliva; and (**D**) transmission efficiency, the proportion of blood-fed mosquitoes presenting virus in their saliva, were tested at 3-, 7- and 14-days post-infection. The error bars are the 95% confidence intervals of the percentages. Number of positive mosquitoes is indicated above each plot. Statistically significant difference between the two CHIKV strain is indicated by asterisks (* *p* < 0.05).

The proportion of infected mosquitoes was low to moderate (<53%) (Figure 1A, Table S1). The infection rates recorded with H2013-445 isolate (ECSA-IOL Lineage) ranged from 7% at 3 dpi to 38% at 14 dpi. For H2019-9293 (Asian lineage), it ranged from 33% at 7 dpi to 53% at 14 dpi (50% at 3 dpi). Infection rates appeared to be higher with the strain H2019-9293 compared to H2013-445. However, a significant difference between the two CHIKV strains was found only at 3 dpi (*p* = 0.0163). Viral strain and dpi were found to be statistically significant predictors of IR (*p* < 0.001 and *p* < 0.05, respectively) (Table 1).

Dissemination rates were relatively high and homogenous between the two viral strains. For the strain H2013-445, even if no dissemination was observed at 3 dpi, dissemination rates were at 83% and 64%, respectively, at 7 and 14 dpi (Figure 1B, Table S1). For the strain H2019-9293, dissemination rates increased from 50% to 100% at 3 and 7 dpi, and decreased to 75% at 14 dpi (Figure 1B, Table S1).

Transmission rates were low and homogenous for the two CHIKV strains ranging from 14% to 17% for the strain H2019-9293 at 3 dpi and 14 dpi, and at 20% for the strain H2013-445 at 7 dpi (Figure 1C, Table S1). The interaction of viral strain and dpi was found to be a significant statistic predictor of TR (*p* < 0.05) but not individually (*p* = 0.1258 and *p* = 0.1703, respectively) (Table 1).

The *Aedes* population from Vientiane capital exhibited low and homogenous transmission efficiency for both CHIKV strains. Among the positive results, values ranged from 3% for H2013-445 at 7 dpi, to 7% for H2019-9293 at 14 dpi (Figure 1D, Table S1).

## 4. Discussion

In Lao PDR, arboviruses represent major public health problems with the circulation or co-circulation of CHIKV, ZIKV, and the four DENV serotypes in Vientiane capital and at the country level [20,21,22,23,26,39]. Intriguingly, despite the evidence of the active circulation of CHIKV for decades in Southeast Asia [15,16,17,18], including in countries bordering Lao PDR, the first CHIKV outbreak was only documented in 2012–2013 in the southern Lao province of Champasak [23,27,28]. The virus at the origin of this epidemic was imported from Cambodia/Thailand and belonged to the ECSA-IOL lineage (23). The high seroprevalence levels observed in rural areas of Champasak province (from 33% to 94%) support active CHIKV transmission by local vectors. An entomological investigation performed at the end of the epidemic indicated the predominance of *Ae. aegypti* among the mosquito larvae collected in Champasak province and it represented 25% of the adults captured, whereas *Ae. albopictus* represented 12% of the larvae and <2% of the adults collected in the same period [23]. However, a few days before this field mission, a vector control campaign was implemented and that could have impacted the population densities reported [23]. In Vientiane capital, *Ae. aegypti* is also the most abundant species representing 86% of the specimens collected between 2016 and 2019 [20]. Interestingly, the 2012–2013 CHIKV outbreak was limited to the south of the country whereas, at the same time, a country-wide DENV-3 outbreak was ongoing [28]. Several studies demonstrated the ability of *Ae. aegypti* to transmit CHIKV but up until now there is no reference data for the local *Aedes* populations [40]. The present study describes and compares for the first time the transmission of different CHIKV strains by *Ae. aegypti,* the predominant vector from Lao PDR.

Our results confirmed that *Ae. aegypti* from Lao PDR can transmit at least two lineages of CHIKV. However, the transmission efficiencies of this population were homogenous, but low for both the ESCA-IOL and Asian lineages (<10%). The results obtained demonstrated a limited infection of our *Ae. aegypti* by CHIKV (<53%), a high dissemination (>50%), and a limited virus transmission (<20%). These observations could emphasize a crucial role of the midgut infection barrier and the salivary gland infection and escape barriers to limit the transmission of this virus for both lineages, whereas no impact was observed for the midgut escape barrier [41,42]. For the infection rate, these results contrasted with previous data obtained for other arboviruses such as DENV-1 and yellow fever virus (YFV; experiments conducted in Institut Pasteur, Paris) in Lao PDR [43,44]. For these flaviviruses, infection rates of *Ae. aegypti* collected in Paksan district, Bolikhamxay province, were high (>70%) [43,44]. Transmission efficiencies appeared to be lower also for CHIKV and *Ae. aegypti* from Vientiane capital (<7%) obtained in this study compare to DENV-1 (50%) [44] but homogenous with YFV (between 0% and 3.2% at 14 dpi) previously obtained with *Ae. aegypti* from Bolikhamxay province [43,44]. These observations highlight the specific genotype-by-genotype interactions between virus and *Ae. aegypti* population and their potential impact on arbovirus emergence and/or outbreak epidemiology through differential virus transmission as previously described for DENV [45,46] and CHIKV [31]. Interestingly, in 2012–2013, during the CHIKV outbreak in southern Lao PDR, a major outbreak of DENV-3 occurred in Vientiane capital and in the rest of the country [22]. Previous studies highlighted the impact of viral competition, during co-infection, on the transmission by *Ae. aegypti* for CHIKV, DENV, and Zika virus [47,48,49]. Due to the endemic and high DENV circulation in Lao PDR, CHIKV circulation could be limited by viral competition in the vector. Evaluation of viral competition in local vectors could increase the characterization of arbovirus transmission in the specific context of Lao PDR.

An evaluation of *Ae. aegypti*‘s ability to transmit CHIKV was also conducted with vector populations from South America and the South Pacific region [50,51,52]. Even if transmission of CHIKV appeared to be high for most of these mosquito populations (>18% after 6 dpi), some populations from South America exhibited a low transmission efficiency as with our *Ae. aegypti* population from Lao PDR. These results demonstrated heterogenous levels of transmission and the importance of studying the interaction between virus and vector for each geographical context to assess the risk of arbovirus emergence. Furthermore, even if the ability of *Ae. aegypti* to transmit CHIKV appeared to be low under laboratory conditions, vector life span as well as its population density could also impact arbovirus emergence as evaluated by the vectorial capacity [53,54]. Vertical transmission is also observed for this virus and is estimated to represent 0.8‰ and 1‰, respectively, in natural and laboratory conditions [55]. This mode of transmission is an important parameter for arbovirus circulation that can be influenced by environmental, taxonomic, and physiological factors, and should be investigated [55].

In Lao PDR, *Ae. aegypti* is considered as the major arbovirus vector [20,24], but *Ae. albopictus* is also recorded as a secondary vector in suburban, rural, and forested areas [25], and other less known species, such as *Aedes malayensis,* are described as potential bridge vectors in sylvatic areas [44]. The presence of these vectors could promote the emergence and the spread of CHIKV specifically in forested areas [56]. Indeed, as previously described, *Ae. albopictus* could transmit CHIKV even if heterogenous transmission efficiency levels were observed in laboratory conditions [50,57]. In Singapore (urban setting), a vector competence study revealed a high infection and dissemination of CHIKV in *Ae. malayensis* [56]. Even if the geographical context is different, the presence of these vectors in rural and forested areas in Lao PDR emphasizes the risk of arbovirus transmission by these secondary vectors, as observed for DENV-1 and its transmission by *Ae. malayensis* from Lao PDR [44]. Furthermore, as demonstrated in France in 2010, a single infected patient could be enough to lead to a CHIKV emergence in the presence of *Ae. albopictus* [58]. The CHIKV transmission by the *Aedes* vector could be influenced also by the presence of amino acid mutation(s) among the CHIKV genome. During the CHIKV outbreak in La Réunion in the Indian Ocean in 2005–2006, a specific mutation (E1-A226V) significantly increased the transmission of the virus by *Ae. albopictus* [31]. In our study, both strains presented this mutation in their genome [23,30]. However, in laboratory conditions, our results demonstrated low transmission efficiencies for both CHIKV strains. During the 2012–2013 outbreak in Lao PDR, some other specific mutations were found among the CHIKV genome and should be investigated to determine if they could modulate the transmission of CHIKV by the local vectors in field conditions [23]. The circulation of CHIKV in Lao PDR and in Southeast Asia could promote the emergence of new mutations in the CHIKV genome and could be followed by better adaptation of the local vectors.

Recently, an active circulation of CHIKV in Southeast Asia was recorded, especially in Thailand, where the ECSA-IOL lineage was detected during a large outbreak in 2019 [59,60]. In Lao PDR, only one CHIKV outbreak was recorded and it was limited to the Champasak province in 2012–2013 [23]. Since then, four imported cases of CHIKV were recorded in Vientiane capital from French Polynesia, Indonesia, Myanmar, and Thailand between 2014 and 2020 without the detection of local circulation in this province [30]. Even if no autochthonous CHIKV cases were detected after the detection of these imported cases, this demonstrated the risk of CHIKV introduction in the country due to presence of the potential vectors *Ae. aegypti* and *Ae. albopictus*. These data emphasize, as demonstrated by the CHIKV and the DENV genetic data recently obtained [20,21,23], not only the active arbovirus circulation between Lao PDR and its neighboring countries but also the rest of Southeast Asia. The same observation was obtained for malaria circulation in southern Lao PDR, where the mobile and migrant population (MMPs), notably involved in forest-related activities, represented a risk of parasite introduction from neighboring countries [61,62]. For several years, the importance of population movement in pathogen circulation represented a challenge to prevent the risk of emerging infectious diseases in this region [63].

Some limitations were recorded during this study. Even if forced salivation is the standard technique used in vector competence studies [50,64,65,66], the saliva volume delivered by the mosquitoes could not be estimated. Therefore, we could have underestimated the transmission rate. Secondly, mosquito rearing conditions and infection protocols were standardized to laboratory settings with controlled temperature and humidity, controlled diet, and larvae density, and a high virus titer in the blood meal. All these parameters could modulate arbovirus transmission [67]. Further investigations, with more natural conditions, should be done on various field-derived populations of *Ae. aegypti* but also on secondary vectors such as *Ae. albopictus* and *Ae. malayensis*.

Taking into account these limitations, all together these data support the risk of the emergence and spread of CHIKV in Lao PDR. Indeed, even if the vector competence of CHIKV seemed to be low in laboratory conditions for the *Ae. aegypti* population tested, several factors could influence the vectorial capacity for this virus and could promote its emergence in Lao PDR. This capacity could be influenced by extrinsic factors such as vector density and lifespan, environmental condition (e.g., temperature and humidity), or population immunity against this arbovirus. It could be also influence by intrinsic factors such as mosquito genetics, immunity, and microbiota [53]. An evaluation of the interactions between the virus, the vector, and the host is essential in order to prevent future outbreaks due to CHIKV in Lao PDR.

## Figures and Tables

**Table 1:** Test statistics of CHIKV infection rate, dissemination rate, and transmission efficiency analyzed by logistic regression. The models included the effect of the viral strain (H2013-445 or H2019-9293), the day post-infection (dpi; 3, 7, or 14), and their interaction.

	**Infection Rate**			**Dissemination Rate**		
	**LR χ^2^**	**Df**	***p* Value**	**LR χ^2^**	**Df**	***p* Value**
Virus	14.81	1	0.0001	2.522	1	0.1123
dpi	9.083	2	0.0107	5.181	2	0.075
Virus × dpi	5.674	2	0.0586	2.488	2	0.2882
	**Transmission rate**			**Transmission efficiency**		
	**LR χ^2^**	**Df**	***p* Value**	**LR χ^2^**	**Df**	***p* Value**
Virus	1.475	1	0.1258	1.475	1	0.2245
dpi	2.197	1	0.1703	2.197	2	0.3333
Virus × dpi	4.578	1	0.0379	4.578	2	0.1014

LR: likelihood ratio; Df: degrees of freedom.

## Data Availability

Not applicable.

## Supplementary Materials

The following supporting information can be downloaded at: www.mdpi.com/article/10.3390/pathogens12010031/s1, Table S1: Infection, dissemination, transmission rates, and transmission efficiency at 3-, 7- and 14-days post-infection (dpi) for the CHIKV tested in this study.
