# Supplementary material for: Low Transmission of Chikungunya Virus by Aedes aegypti from Vientiane Capital, Lao PDR
Source: Pathogens. 2022 Dec 25;12(1):31. doi: 10.3390/pathogens12010031 (PMC9860973; doi:10.3390/pathogens12010031)
Supplement: Supplementary file 1 [file pathogens-12-00031-s001.zip › pathogens-2057763-SI.pdf]

Table S1: Infection, dissemination, transmission rates, and transmission efficiency at 3-, 7- and 14-days post-infection (dpi) for the CHIKV tested in this study.

	**3 dpi**			**7 dpi**			**14 dpi**		
	n	Positive	(%)	n	Positive	(%)	n	Positive	(%)
	**Infection rate (number of infected bodies / number of mosquitoes tested)**								
**H2013-445**	30	2	7	30	6	20	29	11	38
**H2019-9293**	28	14	50	30	10	33	30	16	53
		$p < 0.05$							
	**Dissemination rate (number of infected heads / numbers of infected bodies**								
**H2013-445**	2	0	0	6	5	83	11	7	64
**H2019-9293**	14	7	50	10	10	100	16	12	75
	**Transmission rate (number of infected saliva / numbers of infected heads)**								
**H2013-445**	0	0	0	5	1	20	7	0	0
**H2019-9293**	7	1	14	10	0	0	12	2	17
	**Transmission efficiency (number of infected saliva / number of mosquitoes tested**								
**H2013-445**	30	0	0	30	1	3	29	0	0
**H2019-9293**	28	1	4	30	0	0	30	2	7
